# Radiation hematologic toxicity prediction in rectal cancer: a comparative radiomics-based study on CT image and dose map

**DOI:** 10.3389/fonc.2025.1516855

**Published:** 2025-03-04

**Authors:** Yingpeng Liu, Liping Guo, Yi Wang, Qingtao Xu, Jingfeng Zhang, Xianyun Meng

**Affiliations:** ^1^ Department of Radiology, Ningbo No. 2 Hospital, Ningbo, China; ^2^ Department of Radiotherapy and Chemotherapy, Ningbo No. 2 Hospital, Ningbo, China; ^3^ Cancer Radiochemotherapy Center, First Affiliated Hospital of Ningbo University, Ningbo, China

**Keywords:** dose map, radiomics, radiation hematologic toxicity, CatBoost, rectal cancer

## Abstract

**Background and objectives:**

Acute radiation hematologic toxicity may disturb the radiotherapy plan and thus decrease the treatment outcome. However, whether the dose map has enough prediction value for detecting hematologic toxicity (HT) is still unknown.

**Methods:**

In this study, the pre-treatment CT images and the in-treatment dose map were collected from a discovery dataset of 299 patients and a validation dataset of 65 patients from another center. Then, the radiomic features of the clinical target volume (CTV) in the radiotherapy were extracted, and the least absolute shrinkage and selection operator (LASSO) algorithm was used for feature dimension deduction; three classifiers, that is, support vector machine (SVM) (rbf kernel), random forest, and CatBoost, were used to construct the HT classification model in rectal cancer patients. The model performance was evaluated by both the internal 20% dataset and the external multicenter dataset.

**Results:**

The results revealed that CatBoost achieved the best model performance in almost all tasks and that CT images performed similarly with the dose map, although their combination model performed lower. In addition, gender, age, and some radiomic features from the decomposed image space were the most representative features for HT prediction.

**Conclusion:**

Our study can confirm that the HT occurrence in locally advanced rectal cancer (LARC) patients was multifactorial, and combining effective features together can classify the high-risk patients with HT, thus timely preventing or detecting HT to improve the subsequent outcome.

## 1 Introduction

Radiation hematologic toxicity (HT) is a common complication when locally advanced rectal cancer (LARC) patients are undergoing radiotherapy or chemoradiotherapy (1–3). The appearance of acute HT may disturb the following treatment plan, thus influencing the treatment outcome and survival quality. Therefore, timely recognition of high-risk HT patients is essential for personalized precision treatment of rectal cancer.

In the early years, several studies have been conducted to explore the possibility of HT prediction with the dosimetric factors, and the dosimetric factors of specific regions of interest (ROIs) were consistently reported to be highly associated with HT in rectal cancer (1, 4, 5). However, the commonly used dosimetric factors are simple quantitative metrics, such as V5, V10, and V15, where Vx is the volume percentage with radiation dose more than x Gy. It is clear that the whole dose map contains more dose distribution information than these metrics, but whether the whole dose map can contribute to the HT prediction is still unknown.

In recent years, with the development of radiomics in radiology, the radiomic features that record the subtle characteristics from medical imaging have been widely used for the diagnosis and prognosis of cancer diseases (6–8). Yue et al. first explored the feasibility of pre-treatment CT imaging in HT prediction and revealed that the imaging features performed better than the conventional dosimetric factors (9). Even so, whether the CT imaging maintains better performance in HT prediction than the dose map needs further investigation.

In this study, we designed a comparative study that used a large-sample retrospective LARC dataset that collected both the pre-treatment CT imaging and dose map, which we analyzed using a standardized radiomic procedure. After that, we adopted three different machine learning classifiers to assess the HT prediction performance in both the internal testing dataset and external validation dataset. We infer the dose map may be more sensitive than the CT imaging in HT prediction.

## 2 Methods

### 2.1 Discovery dataset

A total of 336 locally advanced rectal cancer patients from January 2019 to April 2024 were retrospectively included from Ningbo No. 2 Hospital. All patients should satisfy the following criteria: pathologically proven rectal carcinoma, first-time radiation therapy for rectal cancer, and entire blood tests during time points (i.e., within 1 week prior to radiotherapy, once a week during the radiotherapy, and within 1 week after radiotherapy). Patients were excluded if they had 1) a history of radiotherapy or chemoradiotherapy and 2) inconsistent prescription doses, incomplete blood data, or no full course of radiotherapy. A total of 299 patients were finally selected for this study, including 86 patients with acute HT symptoms. **Table 1** provides all patients’ clinical characteristics, such as the clinical stage and cTN stage. This study was approved by the ethics committee of Ningbo No. 2 Hospital. HT was graded according to the Radiation Therapy Oncology Group’s (RTOG’s) acute radiation morbidity scoring criteria. The blood data of all patients were routinely examined 1 week before the radiotherapy and every week during the radiotherapy. Once the patient reached leukocyte grade ≥2, neutrophil grade ≥2, or thrombocytopenia grade ≥2 during radiotherapy, the sample was considered to have HT (9).

**Table 1:** Clinical characteristics of all patients.

Clinical characteristics	Discovery dataset	Validation dataset
Gender (female, male)	77/222	22/43
Age (mean ± SD)	60.42 ± 9.65	58.97 ± 11.32
Clinical stage		
II (T3N0)	41	13
III (T2N2 + T3N2+ T4N0)	258	52
cT stage		
T2	12	6
T3	173	42
T4	114	17
cN stage		
N0	9	2
N1	79	39
N2	203	19
N3	8	5

cT stage, clinical tumor stage; cN stage, clinical lymph node stage; SD, standard deviation.

### 2.2 Validation dataset

To validate the generalization of the HT prediction model, a total of 65 LARC subjects from the First Affiliated Hospital of Ningbo University were also collected using the same inclusion and exclusion criteria, of which 25 patients had symptoms of HT. **Table 1** summarizes the clinical characteristics of the patients, including the clinical stage and cTN stage.

**Figure 1:**
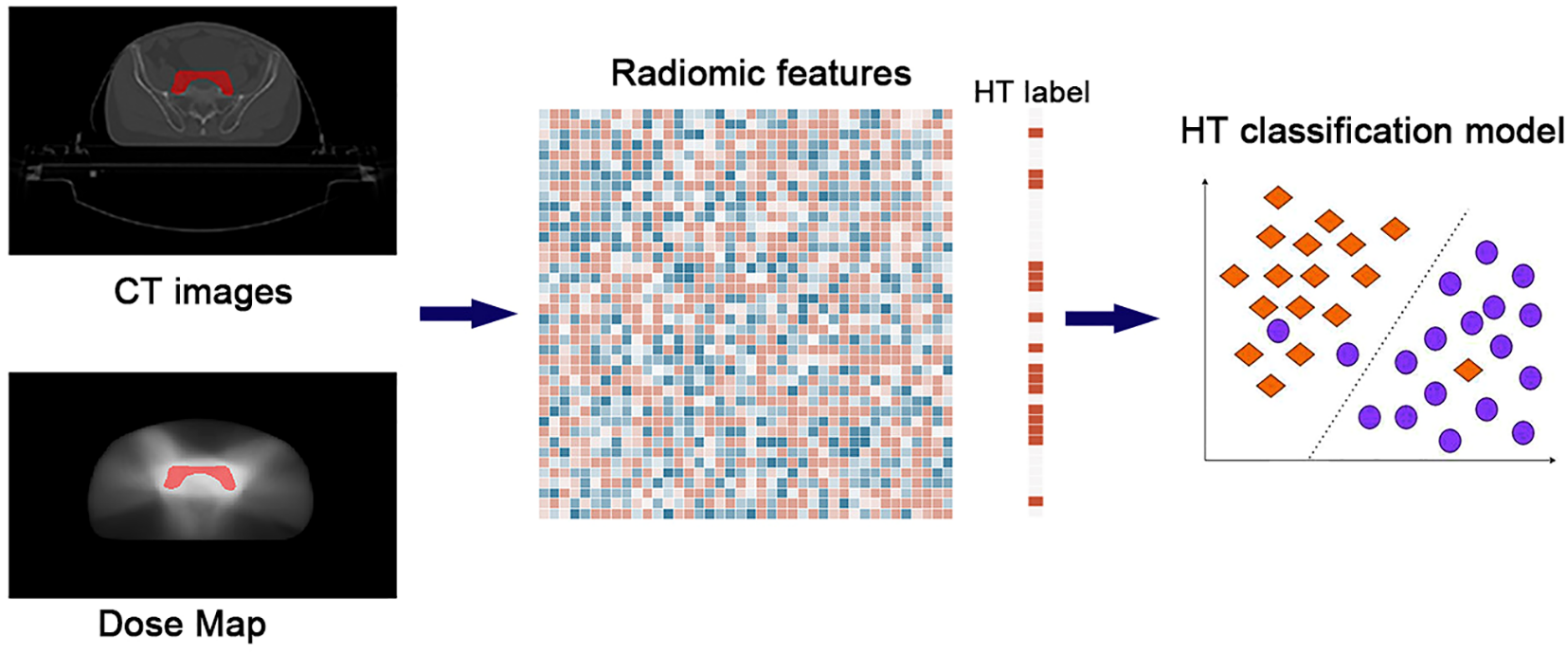
The flowchart of the study. The whole pipeline included three steps: 1) multi-modal data collection, 2) radiomic feature calculation, and 3) machine learning model construction.

The flowchart of this study is illustrated in **Figure 1**, and it mainly includes the following three parts: 1) multi-modal data collection, 2) radiomic feature calculation, and 3) machine learning model construction.

### 2.3 Multi-modal data collection

#### 2.3.1 Pre-treatment CT imaging

The CT images were acquired for simulation and irradiation in subsequent radiotherapy treatment plans by Siemens SOMATOM Sensation Open CT (Siemens, Erlangen, Germany). All patients were in a supine position with a full bladder by drinking 1 L of water 1 hour before the CT scanning, with the following parameters collected: 5-mm thickness, continuous slices without gap, and uniform slice matrix dimension (512 × 512) with voxel size (1.27 mm × 1.27 mm). The validation dataset shared the same scanning parameters as the discovery dataset.

#### 2.3.2 Radiotherapy dose map

In both the discovery and validation datasets, the volumetric modulated arc therapy (VMAT) treatment plans were generated using the Eclipse treatment planning system (V.15.6). All patients received conventional fractionated VMAT with two dose levels simultaneously: 50.6 and 41.8 Gy, 22 or 34 fractions. Treatment was delivered five times per week with a single fraction per day, which was executed on the IX or TrueBeam (Varian Medical Systems, Palo Alto, CA, USA) medical linear accelerator. Finally, every patient would generate a dose map that records the dosimetry distribution at all voxels of abdominal CT images; i.e., the dose map had the same image dimension as the corresponding CT images.

### 2.4 Radiomic feature calculation

In this study, one key ROI used to develop treatment planning was selected for radiomic analysis: clinical target volume (CTV) (10). It was contoured by two experts (Y.L. and L.G.) based on the RTOG guidelines; if there were any disagreements between two experts, a consensus ROI would be obtained by discussion between them. A total of 1,200 radiomic features were extracted using the PyRadiomics software (Version 3.0.1) on treatment planning CT imaging and dose map. The calculated radiomic features were not only from the original image but also from the wavelet-filtered images and Laplacian of Gaussian-filtered images. The type of features mainly included the texture features, such as gray-level co-occurrence matrix, gray-level run-length matrix, gray-level size zone matrix, neighboring gray-tone difference matrix, and gray-level dependence matrix.

### 2.5 Machine learning model construction

In addition to the radiomic features, two demographic (age and gender) features were combined together to construct the HT classification model. Here, only age and gender were selected for the model construction because of their consistently reported roles in HT occurrence (5, 9). To eliminate the dependence on the performance of specific classifiers, three widely used machine learning models were constructed, that is, the support vector machine (rbf kernel), random forest, and CatBoost. All these models were conducted by the scikit-learn package (Version 1.4.2) in Python. Concerning the high-dimensional feature sets, the least absolute shrinkage and selection operator (LASSO) was first used for feature reduction in the training dataset, which was then input into three classifiers to obtain the optimized model. Specifically, the hyperparameters of the LASSO and three classifiers were optimized by an inner fivefold cross-validation in the training dataset. In this study, 80% of the discovery data were used as training data, while the remaining 20% of the data were seen as internal testing data; the multicenter data were deemed as external testing data. The model performance was compared according to accuracy, sensitivity, specificity, and area under the curve (AUC) among the single CT imaging model, the single dose map model, and their combination model.

## 3 Results

**Figure 2:**
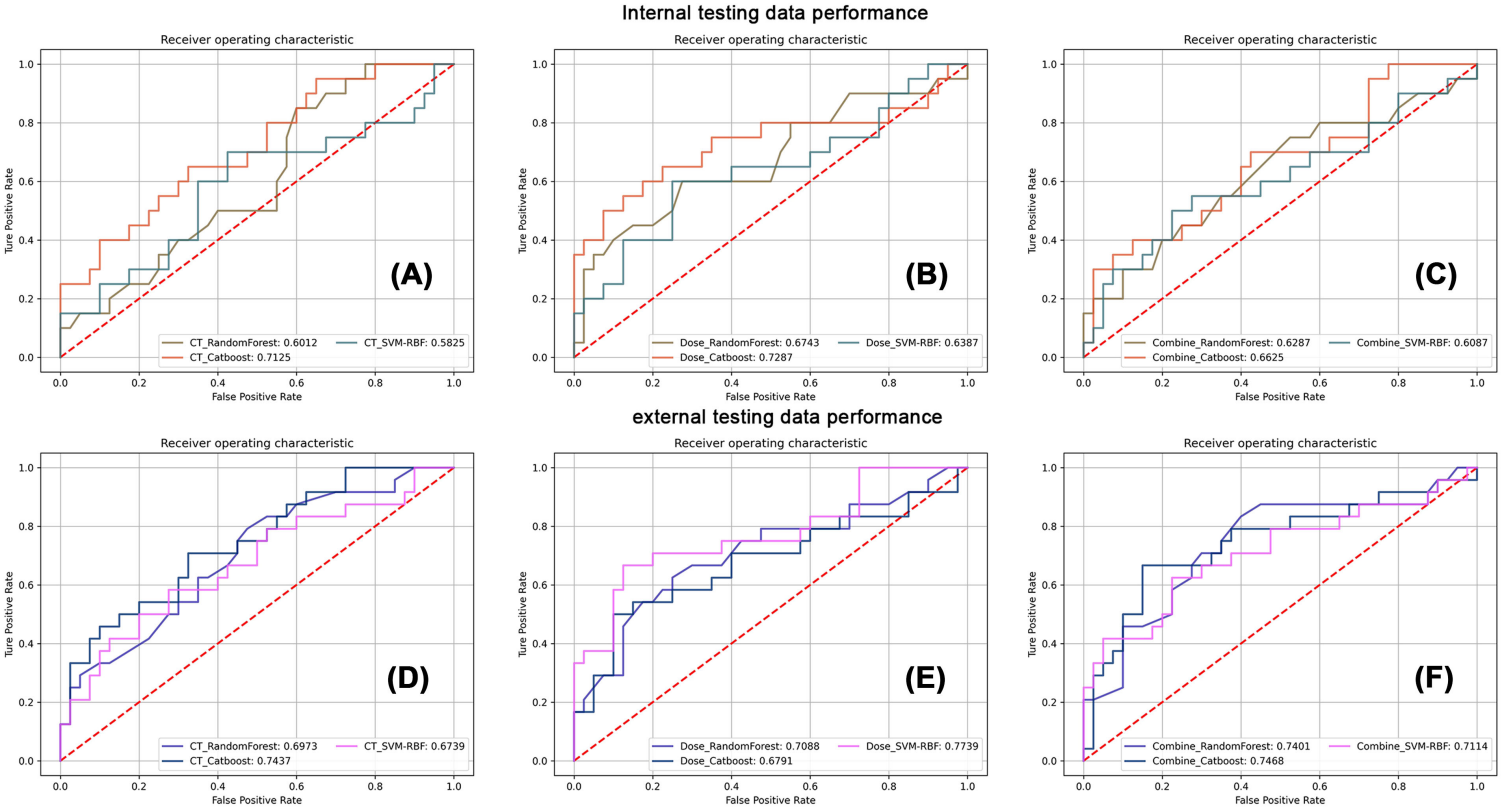
The corresponding receiver operating characteristic analysis of three classifiers in CT imaging, dose map, and their combination models. The first row displays the classification performance of three classifiers in internal testing dataset using CT images **(A)**, the dose map **(B)**, and their combination **(C)**. The second row displays the classification performance of three classifiers in external testing dataset using CT images **(D)**, the dose map **(E)**, and their combination **(F)**.

**Figure 2** summarizes the HT model performance of three different classifiers, and the CatBoost model achieved the best AUC in almost all conditions. The dose map performed similarly with the CT imaging in HT prediction, while their combination had lower performance. **Table 2** shows the detailed performance metric of every classifier in all groups, and it should be emphasized that the specificity in these models was not high enough, which may explain the current challenge in HT prediction.

**Table 2:** The model performance of different classifiers in HT prediction.

Classifier	CT—internal testing dataset				CT—external testing dataset			
	Accu (%)	Sen (%)	Spec (%)	AUC (%)	Accu (%)	Sen (%)	Spec (%)	AUC (%)
RF	60	75	30	60.1	64.1	77.5	41.7	69.7
CatBoost	**68.3**	**75**	**55**	**71.2**	**68.8**	**80**	**50**	**74.4**
SVM-rbf	58.3	72.5	30	58.2	68.8	87.5	37.5	67.4
	Dose—internal testing dataset				Dose—external testing dataset			
	Accu (%)	Sen (%)	Spec (%)	AUC (%)	Accu (%)	Sen (%)	Spec (%)	AUC (%)
RF	71.7	97.5	20	67.4	65.6	87.5	29.2	70.9
CatBoost	**78.3**	**100**	**35**	**72.9**	68.8	90	33.3	67.9
SVM-rbf	70	95	20	63.9	**75**	**90**	**50**	**77.4**
	Combination—internal testing dataset				Combination—external testing dataset			
	Accu (%)	Sen (%)	Spec (%)	AUC (%)	Accu (%)	Sen (%)	Spec (%)	AUC (%)
RF	68.3	90	25	62.9	73.4	90	45.8	74
CatBoost	**71.7**	**90**	**35**	**66.2**	**71.9**	**90**	**41.7**	**74.7**
SVM-rbf	71.7	92.5	30	60.9	73.4	92.5	41.7	71.1

RF, random forest; Accu, accuracy; Sen, sensitivity; Spec, specificity; AUC, area under curve; SVM, support vector machine. The bold font means the best performance among three classifiers.

**Figure 3:**
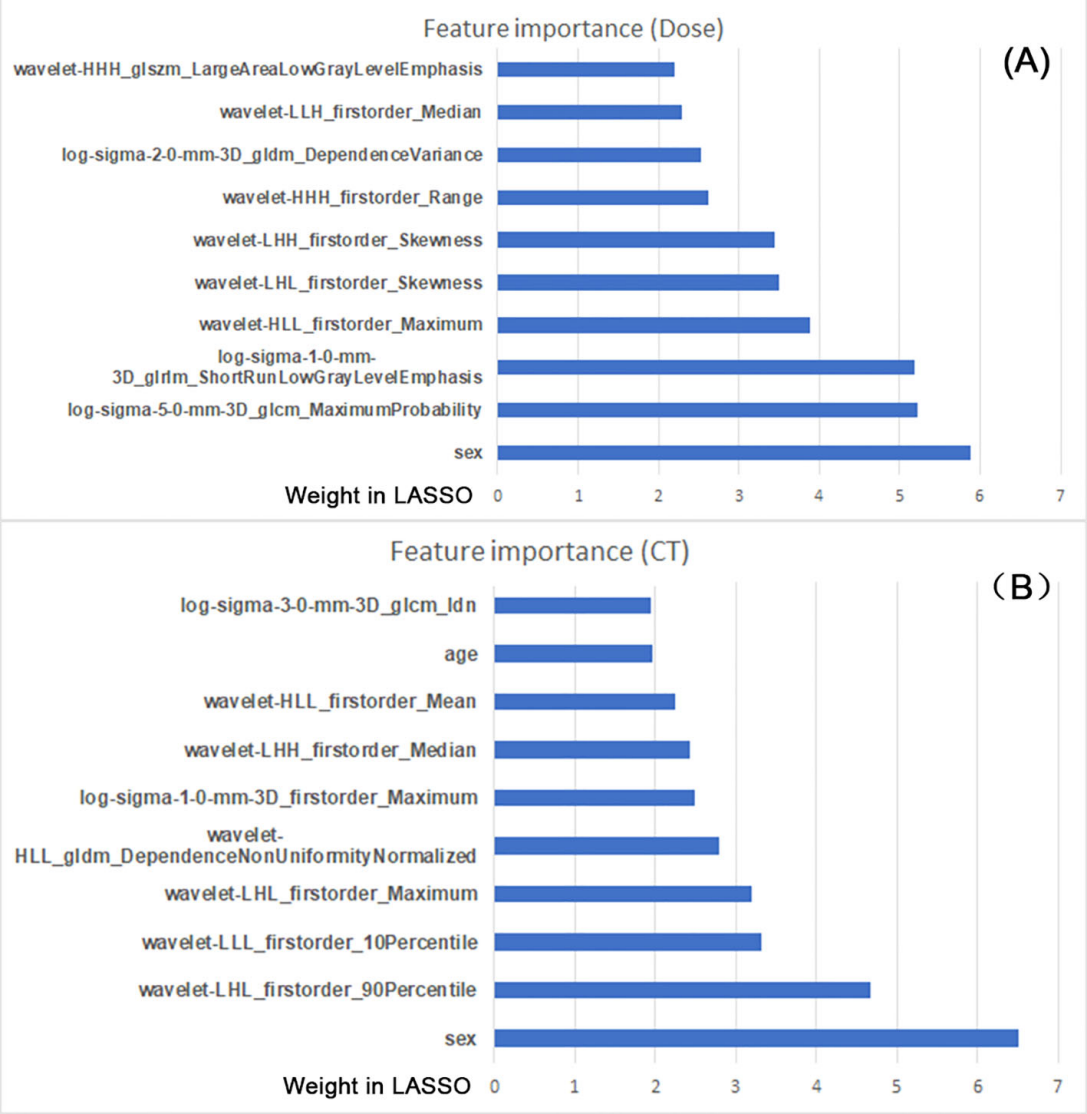
The top 10 features from the constructed models used in the CT images **(A)** and dose map **(B)**. The feature importance was sorted by the weight coefficient in LASSO model (X-axis); the feature names are listed (Y-axis).

**Figure 3** listed the top 10 features used in the CT imaging and dose map models, and sex was found to be a key demographic factor in both groups. In addition, age was related to HT prediction in the CT imaging model. In addition to the demographic features, the other features were mainly from the texture features of wavelet-transformed images, especially the type of first-order features.

## 4 Discussion

In this study, we compared the HT prediction performance using the radiomic features of CTV in two imaging modalities with three classifiers and found that single dose map performed similarly with single pre-treatment CT images, but their combination performed worse than any single one. In addition, the model performance was dependent on the selection of the classifier, and sex is a key demographic feature in HT prediction. Our study can strengthen the understanding of the contribution factors to HT occurrence; i.e., the initial status of rectal cancer (CT images) and the dosage prescription (dose map) are both effective indicators, which may shed light on the timely diagnosis of high-risk HT patients and thus improve the subsequent individualized treatment response.

However, precise prediction of HT in rectal cancer patients during radiotherapy is still challenging. Our study revealed that the pre-treatment CT imaging performed similarly to the dose map of radiotherapy. The phenomenon may indicate that HT’s existence was related to multiplex factors; both pre-treatment status and radiotherapy treatment plan may contribute to HT. Even so, the classification accuracy was still not high; we infer that there may be some other latent factors that may influence the model performance, such as the treatment regimen (11), although tumor heterogeneity may be another invisible factor. Compared to a previous study (9), our study emphasized that using the dose map instead of the dosimetric features could be better for HT prediction. Especially, the previous study adopted eight different ROIs for HT prediction, and the most sensitive ROI was bone marrow, while CTV performed relatively low. In contrast, our study only used the tumor-related CTV as ROI, and its performance was acceptable in both CT imaging and dose map groups. A feasible interpretation was that we utilized three classifiers, which may have decreased the performance dependence of a single classifier in a previous study.

In this study, only two demographic features were included in the HT prediction model. On the one hand, age and gender are usually collected for LARC patients in the clinical practice of our hospital, while the other demographic information was not available for every patient. On the other hand, age and gender have been consistently reported in the HT prediction model (5, 9), and the main purpose of this study was to ascertain whether the dose map also contributes to HT prediction like CT imaging. In this study, gender was found as a key demographic feature for HT classification in both CT imaging and dose map models, implying that the HT occurrence was selective to gender in the initial tumor status (CT image) and the treatment plan (dose map). In our population, the sample size of male LARC patients was obviously larger than that of female patients, while the existence ratio of HT was higher in female patients. Additionally, the CT images also revealed that age was also an important factor, thus suggesting the pre-treatment factors were dependent on both gender and age. These findings add to the knowledge about the course of HT for LARC patients, implying that multi-modal fusion may serve as the essential avenue for improving HT prediction.

In addition to the demographic features, the radiomic features were also highly associated with the HT occurrence. Radiomic features reflect the microscopic characteristics within images (12, 13), and different imaging modalities adopted specific features, indicating that CT images and dose map had different participating roles in HT. Moreover, most features were from the wavelet transformed and Laplacian of Gaussian filtered images, again prompting that the most informative features were not from the original images. In addition, when combining the features of two modalities together, the model performance was slightly decreased, and this may be explained by the correlative features between the two modalities.

Finally, three different classifiers displayed a non-consistent tendency in HT classification. CatBoost performed the best in comparison to support vector machine-rbf (SVM-rbf) and random forest in this study. CatBoost is composed of gradient-boosted decision trees and shows low risk for overfitting, robustness to outliers, and fast training speed. In previous studies, it had also achieved good model performance in different tasks using radiomic features (7, 8, 14). In contrast, SVM-rbf and random forest performed clearly worse than CatBoost, although they are also widely used machine learning classifiers. This phenomenon underscores the essentials of selecting multiple classifiers in radiomic studies, which may more effectively unveil the application significance of machine learning models in clinical diagnosis.

Several limitations were available in this study: 1) we only compare the radiomic model of one ROI (CTV), and some other ROIs may be also sensitive to HT classification, which needs further studies in the future. 2) The models’ performance may be also influenced by the feature selection manner, and we only utilized the LASSO, while other feature selection algorithms may perform better. 3) The main purpose of this study is not to improve the HT prediction performance using radiomics but to confirm whether the dose map contributes to HT prediction like CT images, so there are many other unexplored factors that may be also important for HT occurrence, which can be explored in the future. 4) The dose intensification in rectal cancer (15) may be also influential to HT, which can be explored in future studies.

## 5 Conclusion

In this study, we found the radiomic features of CTV from the dose map could be used for HT prediction in LARC patients like CT images, although their combinations were not good enough. In addition, sex and age were also related to HT occurrence, and CatBoost gained the best model performance. Our study could deepen the understanding of the contributing factors and their complex interacting roles in HT prediction, which may be helpful to timely detect high-risk HT patients and make a targeted treatment strategy for these patients.

## Data Availability

The raw data supporting the conclusions of this article will be made available by the authors, without undue reservation.
